# Evaluating the influence of life‐history characteristics on genetic structure: a comparison of small mammals inhabiting complex agricultural landscapes

**DOI:** 10.1002/ece3.2269

**Published:** 2016-08-18

**Authors:** Elizabeth M. Kierepka, Sara J. Anderson, Robert K. Swihart, Olin E. Rhodes

**Affiliations:** ^1^ Savannah River Ecology LaboratoryUniversity of GeorgiaPO Drawer EAikenSouth Carolina29802; ^2^ Biosciences DepartmentMinnesota State University Moorhead1104 7th AveMoorheadMinnesota56563; ^3^ Department of Forestry and Natural ResourcesPurdue University715 W. State StreetWest LafayetteIndiana47907

**Keywords:** Comparative landscape genetics, ecological specialization, fragmentation, Upper Wabash Valley

## Abstract

Conversion of formerly continuous native habitats into highly fragmented landscapes can lead to numerous negative demographic and genetic impacts on native taxa that ultimately reduce population viability. In response to concerns over biodiversity loss, numerous investigators have proposed that traits such as body size and ecological specialization influence the sensitivity of species to habitat fragmentation. In this study, we examined how differences in body size and ecological specialization of two rodents (eastern chipmunk; *Tamias striatus* and white‐footed mouse; *Peromyscus leucopus*) impact their genetic connectivity within the highly fragmented landscape of the Upper Wabash River Basin (UWB), Indiana, and evaluated whether landscape configuration and complexity influenced patterns of genetic structure similarly between these two species. The more specialized chipmunk exhibited dramatically more genetic structure across the UWB than white‐footed mice, with genetic differentiation being correlated with geographic distance, configuration of intervening habitats, and complexity of forested habitats within sampling sites. In contrast, the generalist white‐footed mouse resembled a panmictic population across the UWB, and no landscape factors were found to influence gene flow. Despite the extensive previous work in abundance and occupancy within the UWB, no landscape factor that influenced occupancy or abundance was correlated with genetic differentiation in either species. The difference in predictors of occupancy, abundance, and gene flow suggests that species‐specific responses to fragmentation are scale dependent.

## Introduction

The negative impacts of human‐induced fragmentation are numerous (Andrén 1994; Fahrig 2003; Cushman 2006), and sensitivities to such impacts vary greatly even within codistributed species (e.g., Andrén 1994; Swihart et al. 2003; Rizkalla and Swihart 2006; Meyer et al. 2008; Bommarco et al. 2010; Lange et al. 2010). Species’ responses to landscape change ultimately depend on traits such as ecological specialization, dispersal ability, body size, and population size because these factors dictate how well species can persist in patches as well as their ability to colonize new patches (Hanski 1998; Etienne and Heesterbeck 2001). Fragmented populations can suffer from lower effective population sizes and decreased population connectivity, which in turn lead to losses in genetic diversity (e.g., Johansson et al. 2007; Dixo et al. 2009; Vranckyx et al. 2012; Méndez et al. 2014). Erosion of genetic diversity increases the risk of inbreeding, genetic drift, and decreased evolutionary potential, all of which increase the probability of extirpation (Young et al. 1996; Saccheri et al. 1998; Reed and Frankham 2003). As a result of the potential negative consequences of fragmentation, considerable attention has focused on identifying species traits that increase vulnerability to fragmentation (e.g., Swihart et al. 2003; Henle et al. 2004; Ewers and Didham 2006; Barbaro and Van Halder 2009; Blanchet et al. 2010) and investigating potential mitigation techniques (e.g., McRae et al. 2012; Breckheimer et al. 2014; Tambosi et al. 2014).

In the large body of literature on species‐specific responses to habitat loss and fragmentation, several ecological factors known to increase species sensitivity to the negative impacts of habitat loss and fragmentation have been identified (e.g., Swihart et al. 2003; Rytwinski and Fahrig 2012; Jauker et al. 2013; Newbold et al. 2013; Slade et al. 2013; Newmark et al. 2014). Among these traits, body size and ecological specialization are frequently cited as being correlated with vulnerability to fragmentation (e.g., Swihart et al. 2003; Watling and Donnelly 2007; Bommarco et al. 2010). Body size is correlated to many life‐history traits known to affect species’ responses to fragmentation, including dispersal ability, geographic range, and reproductive rate (e.g., Blueweiss et al. 1978; Lindstedt et al. 1986; Hernández Fernández and Vrba 2005; Jenkins et al. 2007). In general, larger species are expected to have greater dispersal capabilities (Whitmee and Orme 2013), perceptual ranges (Mech and Zollner 2002), and geographic ranges (Diniz‐Filho and Tȏrres 2002; Hernández Fernández and Vrba 2005), so they can more easily colonize new patches of suitable habitat than can smaller species. Larger species, however, have low reproductive rates and require large home ranges, which can increase extirpation risks (e.g., Cardillo et al. 2005; Rytwinski and Fahrig 2012). Therefore, the role of body size in predicting how species respond to habitat alteration is not always clear (Henle et al. 2004; Ewers and Didham 2006), especially when codistributed species of similar size display different degrees of ecological specialization.

Independent of body size, ecological specialization limits the amount of habitat a species can occupy as well as the number of potential routes they can utilize to travel between patches. Consequently, specialists are expected to be highly sensitive to habitat loss and fragmentation. Empirical data have largely supported this expectation because specialist species tend to have lower abundances or occupy fewer patches within fragmented landscapes (e.g., Berglund and Jonsson 2008; Devictor et al. 2008; dos Anjos et al. 2011). Habitat loss and fragmentation are also expected to reduce connectivity between patches in specialists and thus contribute to genetic discontinuity. Patterns of genetic differentiation, however, vary considerably across specialists (e.g., Exeler et al. 2008; Bommarco et al. 2010; Brückmann et al. 2010; Lawton et al. 2011; Gil‐López et al. 2014; Ripperger et al. 2014; Berkman et al. 2015), so predicting how any one trait will impact genetic responses to fragmentation is difficult.

To examine how body size and ecological specialization influence gene flow across fragmented landscapes, we conducted our study within the Upper Wabash River Basin (UWB), Indiana, USA. The UWB has been largely converted to agriculture and has been subject of extensive study to assess the ecological effects of land conversion on a variety of vertebrate species (Nupp and Swihart 1998, 2000; Goheen et al. 2003; Swihart et al. 2003; Moore and Swihart 2005; Dharmarajan et al. 2009; Beatty et al. 2012; Anderson et al. 2015). These studies have confirmed that both landscape heterogeneity and life‐history traits, particularly ecological specialization, influence patterns in occupancy and abundance (Nupp and Swihart 1998; Swihart et al. 2003; Moore and Swihart 2005; Beasley et al. 2015). In rodents, for example, dependence on forest largely predicted if a species was sensitive to habitat fragmentation, whereas generalist species tended to benefit from agriculture regardless of body size (Nupp and Swihart 1998, 2000; Swihart et al. 2003). Building on the previous studies on forest‐associated rodents, we aimed to elucidate whether traits that affected occupancy and abundance in forest rodents also predicted patterns in gene flow across the UWB.

Our focal species, the eastern chipmunk (*Tamias striatus*) and white‐footed mouse (*Peromyscus leucopus*), are largely ubiquitous within the UWB, but are expected to exhibit contrasting patterns in gene flow based on their different life histories. Chipmunks are larger than white‐footed mice and are known to move farther distances (Rizkalla and Swihart 2007), so chipmunks may be able to traverse potential barriers (i.e., unsuitable habitat) more readily than white‐footed mice. Under this scenario, chipmunks would experience high gene flow within the UWB and exhibit weak genetic structure as compared to white‐footed mice. However, chipmunks are considered more dependent on forested habitats than white‐footed mice, which in the highly fragmented UWB, may limit their ability to cross unsuitable habitats. Suitable forest habitat certainly influences chipmunk fine‐scale gene flow (Anderson et al. 2015), occupancy (Moore and Swihart 2005), and simulated abundance (Rizkalla and Swihart 2012) in the UWB, so forest habitat may impact chipmunk genetic structure across the UWB as well. In contrast, white‐footed mice are generalists, so their willingness to utilize alternative habitats may result in higher gene flow and corresponding weaker genetic structure than chipmunks in the UWB. Furthermore, forested habitat had a weak impact on occupancy and abundance within the UWB, so white‐footed mice may not exhibit gene flow that is correlated with forested habitats such as chipmunks.

## Material and Methods

### Study area

**Figure 1:**
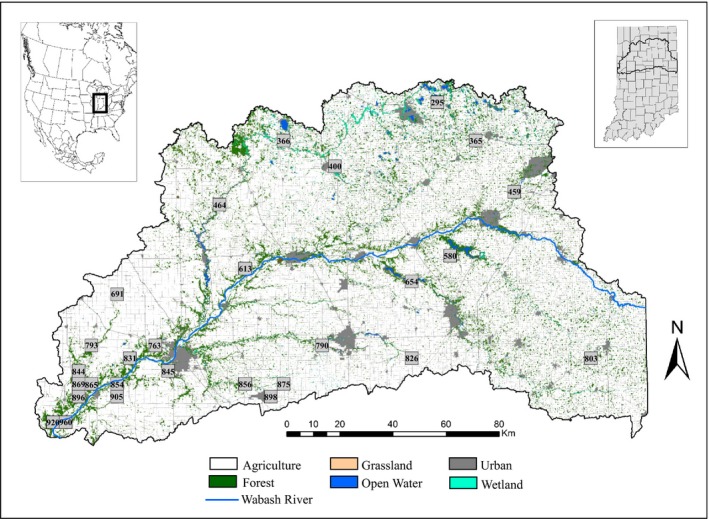
Distribution of 28 study cells across the Upper Wabash Basin (UWB), Indiana, where trapping for eastern chipmunks and white‐footed mice occurred from 2001 to 2003. Land cover within the study area is primarily agriculture with forests along river tributaries. The Wabash River (blue online, gray in print) runs east–west across the entire study area.

Our study area encompassed the Upper Wabash River Basin (UWB; Fig 1) in north‐central Indiana, USA. The UWB contains eight major watersheds that cumulatively drain greater than 20% of the state (>20,000 km^2^; Swihart and Slade 2004). Prior to European settlement, much of the UWB was forested (87% statewide; Smith et al. 1994), but conversion to agriculture has reduced forest cover to 8% within UWB. The remaining forests (mainly *Quercus–Carya–Acer*) are highly fragmented and tend to be clustered around the major drainages within UWB because floodplains or topography was not suitable for agriculture. Currently, 96% of UWB is privately owned with 88% designated as agriculture.

### Sample collection

A full description of sampling methods can be found in Moore and Swihart (2005) and Urban and Swihart (2009), but briefly, trapping for eastern chipmunks and white‐footed mice occurred within 35, 23 km^2^ study sites (hereby called study cells) in the UWB during the summers of 2001–2003 (Fig. 1). Study cells were selected via algorithms designed to maximize the diversity of land cover types sampled within the UWB (Urban and Swihart 2009). Within each study cell, potential locations for trapping grids (30 × 30 m pixels) were classified according to 1 of 5 land cover categories (agriculture, forest, grassland, wetland, or urban) using geographic information system layers of land use (Moore and Swihart 2005; Urban and Swihart 2009). We then used a stratified random design to select trapping grid locations within study cells so that natural land cover types (i.e., grassland, forest, and wetland; 27.8% of grids for each land cover type within study cells) were disproportionally represented as compared to urban and agriculture (13.9 and 2.8% of all grids within study cells, respectively). In total, a maximum of 45 trapping grids were placed within each study cell with 1–3 grids within a patch of habitat (Swihart and Slade 2004; Moore and Swihart 2005; Urban and Swihart 2009).

Trapping methods within study cells (i.e., grid dimensions and type of trap) varied by year and forest patch size. In 2001, we placed either 3 × 3 (small to medium forest patches) or 7 × 7 (large forest patches) grids of Fitch live traps spaced 15 m apart within study cells. Sherman live traps or a mix of Fitch and Sherman live traps, arranged as 3 × 3 or 5 × 5 trapping grids, was deployed in study cells in 2002 and 2003. Habitat corridors identified as treed and nontreed land cover features <30 m wide were fitted with 5 × 2 grids. Each trapping session consisted of a prebait period with traps locked open for 3 days, followed by a 5‐day trap‐check session, during which traps were checked twice daily. Traps were baited with black oil sunflower seeds, and upon capture, ear or toe clips were removed from animals using sterile scissors. Following sampling, animals were treated with ferric subsulfate if bleeding occurred and released. All animals were handled according to procedures approved by the Purdue Animal Care and Use Committee under protocol #01–024. We stored all tissues at −80°C until DNA extraction.

### Microsatellite genotyping

We used an ammonium acetate protocol and an ethanol wash to extract DNA from tissue samples (modified from the PUREGENE kit; Gentra Systems, Minneapolis, MN). DNA quality was checked by running 3 *μ*L of DNA out on a 2% agarose gel stained with ethidium 12 bromide, and then, DNA extracts were diluted to a final concentration of approximately 10 ng/*μ*L. Chipmunk samples were amplified at 12 (EACH01‐12; Anderson et al. 2007) microsatellite loci, while white‐footed mice were amplified at 10 loci [PO‐26, PO‐85, Po‐97 (Prince et al. 2002); Pml01, Pml02, Pml05, Pml09, Pml12 (Chirhart et al. 2000); PLGT15 (Schmidt 1999)]. Amplification by multiplex PCR took place in 10 *μ*L volumes with 20 ng of template DNA, 0.2 mM of each dNTP, 1 U of *Taq* DNA polymerase (NEB), and 2× Thermopol reaction buffer (20 mM Tris‐HCl, 10 mM (NH_4_)_2_SO_4_, 10 mM KCl, 2 mM MgSO_4_, 0.1% Triton X‐100; NEB). The amount of primer for each locus (0.05–0.30 *μ*M) was adjusted so that all loci in the multiplex reaction would result in approximately equal intensities of product. The amplification conditions were as follows: initial 94°C for 2 min, 35 cycles of 94°C for 30 s, primer‐specific annealing temperature for 30 s, 72°C for 30 s, then a final extension of 72°C for 10 min, and a soak at 60°C for 45 min. The PCR products were sized on an Applied Biosystems 3730 automated sequencer, and the genotypes were determined for all loci in all individuals using the software GeneMapper 3.7 (Applied Biosystems, Foster City, CA).

We utilized multiple quality control methods to ensure accuracy of microsatellite genotypes. First, a negative control, two pre‐amplified positive controls, and a concurrently amplified positive control were run on every 96‐well plate. Any ambiguous samples were re‐amplified and genotyped again at all loci, and any missing genotypes were re‐amplified in the multiplex reaction up to two times. If there were still missing genotypes after re‐amplifying the multiplex, samples were amplified using single locus reactions to attempt to retrieve the missing genotypes. Finally, if missing genotypes remained, we re‐extracted the samples and genotyped the individuals at all loci. Only individuals with <30% missing genotypes were accepted into the final dataset. Following quality control, we removed 7 study cells that occurred within the eastern portion of the UWB from our dataset because they did not provide sufficient sample sizes of one of the species for genetic analysis. Our final dataset consisted of 1229 chipmunks (107 of 14,916 or 0.717% missing genotypes) and 959 white‐footed mice (237 of 9630 or 2.461% missing genotypes) across 28 study cells.

### Population differentiation and diversity

For each study cell, deviations from Hardy–Weinberg (HWE) and linkage equilibrium (LE) were calculated in genepop (Raymond and Rousset 1995). We corrected for multiple tests using false discovery rate (*α *= 0.013; Benjamini and Yekutieli 2001), and null allele frequencies were calculated in micro‐checker (Van Oosterhout et al. 2004). Microsatellite loci were eliminated from analyses if they consistently deviated from HWE in all study cells. We then used the R (R Development Core Team 2013) package diveRsity (function “divBasic”; Keenan et al. 2013) to quantify four genetic diversity measures (allelic richness, number of alleles, heterozygosity, and *F*_IS_). Two measures of genetic differentiation between study cells, *F*_ST_ and Jost's *D* (*D*_EST_; Jost 2008), were also calculated in diveRsity to serve as response variables for the landscape genetic analyses. For each genetic diversity and differentiation metric, 95% confidence intervals were calculated after 10,000 permutations.

To gain insight into potential major barriers to gene flow within the entire UWB study area, we tested two potential causes (geographic distance and the Wabash River) using Mantel tests. First, a simple Mantel test quantified the relationship between geographic distance and genetic differentiation; a positive relationship would indicate the presence of isolation‐by‐distance (IBD; Wright 1943). Second, we utilized a partial Mantel test to test whether the Wabash River, the major river within our study area, serves as a barrier to gene flow. A partial Mantel test allows for control of variables such as geographic distance and hence isolation of the variable of interest (i.e., Wabash River). Pairs of individuals were coded as either a 1 (different sides of the Wabash River) or a 0 (same side) for the partial Mantel test. All Mantel test calculations were performed in the R package *vegan* (Oksanen et al. 2012) using the functions “mantel” and “mantel.partial” via 10,000 permutations.

In addition to the UWB landscape‐level tests for IBD and isolation due to the Wabash River, we also tested for the presence of IBD within each 23 km^2^ study cell using individual‐based simple Mantel tests. Individual‐based genetic distances (Rousset's *a*; Rousset 2000) were calculated in spagedi v. 1.2 (Hardy and Vekemans 2002), and simple Mantel tests were performed in *vegan*. As a complement to the simple Mantel tests, we tested for fine‐scale genetic structure using spatial autocorrelations in genalex 6.2 (Peakall and Smouse 2006). Both species were expected to exhibit significant positive spatial autocorrelation coefficients (*r*; Peakall et al. 2003) at small distance intervals due to restricted dispersal. We evaluated different distance intervals (50–500 m) as recommended by Banks and Peakall (2012) and found 100‐m intervals maintained a sufficient sample size within each distance interval for both species. Regardless of the distance interval, we bounded our analyses between 0 and 2 km in spatial autocorrelation calculations.

### Bayesian clustering analysis

To identify the number of genetic clusters of chipmunks and white‐footed mice within the UWB, we utilized two Bayesian clustering programs: structure (Pritchard et al. 2000) and baps 6 (Corander et al. 2008). Both programs group individuals into predefined genetic clusters (*K*) that minimize deviations from HWE and LE. While these programs have been shown to be robust under a number of potential scenarios (e.g., Latch et al. 2006; Safner et al. 2011), multiple authors suggest running several Bayesian programs to examine variability according to calculation methods and prior information (Latch et al. 2006; Frantz et al. 2009).

structure was performed under two scenarios: one with no prior spatial information and one with study cell included under the LOCPRIOR option. All Bayesian programs including structure can identify false clusters due to weak barriers to gene flow (Latch et al. 2006) or sampling along a genetic gradient (Frantz et al. 2009; Schwartz and McKelvey 2009), so including a spatial prior helps to minimize detection of erroneous clusters. For each scenario, we initially tested *K *=* *1–20 with 10 replicate runs (100,000 MCMC burn‐in, 100,000 permutations) at each *K* under the admixture, correlated alleles model. The optimal *K* was then determined via the Evanno et al. (2005)'s Δ*K* method, and ten longer runs (1,000,000 MCMC burn‐in, 1,000,000 permutations) at the optimal *K* were used to calculate *q*‐values, the proportion of each individual's genome that belongs to each cluster. *Q*‐values were averaged across the ten longer runs via the program clumpp (Jakobsson and Rosenberg 2007), and individuals were assigned to a cluster based on their highest *q*. Each cluster was then run iteratively using the same conditions as the full dataset to identify any hierarchical structure across the UWB. We continued iterative runs until all clusters had no further structure as indicated by Δ*K*.

For comparative purposes, we also employed a spatially explicit approach in baps 6 to identify genetic structure of chipmunks and white‐footed mice across the UWB. baps utilizes geographic information (i.e., geographic coordinates) as a prior, and based on maximum likelihood and highest posterior probabilities, determines the optimal *K*. We tested *K *=* *1 through 20 with 10 replicates per *K* using the “Spatial Clustering of Individuals” option and saved the output for the admixture analysis. Admixture between inferred clusters was calculated using 500 simulations based on observed allele frequencies.

### Landscape genetics: landscape configuration between study cells

Gene flow in both species is likely a function of both landscape configuration between study cells and landscape complexity within study cells (Pflüger and Balkenhol 2014). Previous studies have documented that landscape configuration between populations impact gene flow in both chipmunks (Anderson et al. 2015) and white‐footed mice (Munshi‐South 2012), so we expected configuration to be correlated with genetic differentiation in both species. Within the UWB, chipmunks and white‐footed mice are fairly ubiquitous (Moore and Swihart 2005), but multiple measures of landscape complexity within study cells have been related to variance in abundance (Rizkalla and Swihart 2012) and occupancy (Moore and Swihart 2005) within the study area. Therefore, landscape genetic hypotheses incorporated both landscape configuration between study cells and metrics of landscape complexity that have previously been correlated with abundance and occupancy within the UWB.

To evaluate how landscape configuration between study cells could influence gene flow, we designed six resistance surfaces for each species based on the 30 × 30 m national land cover database (NLCD 2001; Homer et al. 2007) raster clipped to the UWB. The first two resistance surfaces, isolation‐by‐distance (IBD) and isolation‐by‐barrier (IBB), served as null hypotheses. All pixels within the IBD resistance surface were given a value of 1, and the IBB resistance only assumed that open water was highly resistant to movement. Consequently, the resistance of open water in the IBB resistance surface was set to 500 with all other pixels set to 1.

The remaining four resistance surfaces were parameterized using species‐specific movement and mortality data derived from six land cover types common in the UWB (forest, wetland, urban, open water, grassland, and agriculture; Rizkalla and Swihart 2012; Table S1).

For each of these four resistance surfaces, forest was assumed to be the preferred habitat of both species and thus was assigned a resistance value of 1 (probability of mortality = 0.01, movement = 1.0; Rizkalla and Swihart 2012). Resistances for all other land cover types were calculated based on their probabilities of mortality or movement defined in Rizkalla and Swihart (2012; Table S1). For example, the probability of a chipmunk moving into wetland in Rizkalla and Swihart (2012) was five times lower than forest, so the resistance value for wetland was 5 for the movement surfaces. Unlike all other land cover types, we had to combine roads and urban habitat into a single category (urban) due to the spatial extent of our study area. Combining these categories presented a potential problem because while urban habitat and roads are known to impede gene flow in rodents (e.g., Munshi‐South 2012; Marrotte et al. 2014), mortality and movement probabilities (and by extension resistance values) were much higher for roads than unroaded urban habitat (Rizkalla and Swihart 2012). To reconcile the differences between roaded and urban habitats, we varied resistance for urban to reflect either resistances of roads or urban habitat as defined in Rizkalla and Swihart (2012; Table S2). Thus, each species had two null hypothesis surfaces (IBD and IBB), two based on urban mortality (high resistance for urban = MortH, low resistance for urban = MortL), and two based on urban movement probabilities (high resistance for urban = MoveH, low resistance for urban = MoveL).

Each of the six resistance surfaces (IBD, IBB, MortL, MortH, MoveL, and MoveH; Table 1) was used as an input to the program circuitscape v 4.0.5 (McRae and Shah 2009) to calculate landscape resistance distances between study cells. Resistance distances, in essence, represent the difficulty of traversing the landscape between study cells, so resistance distances are expected to be positively correlated with genetic differentiation between study cells. Calculation methods in circuitscape combine graph and circuit theory by constructing a graph of all the study cells where each study cell is a node connected through edges (i.e., potential dispersal paths between study cells). Edges then function as resistors on an electrical circuit where the magnitude of each resistor can be defined by the resistance surface provided. Resistance distances between study sites for a given resistance surface are calculated by summing all resistors (i.e., edges between study cells) across all possible pathways. Multiple pathways are more realistic than single path analyses (e.g., least cost path; McRae and Beier 2007) because neither of these rodent species is likely to utilize a single path for dispersal.

**Table 1:** Landscape variables for the landscape genetic analysis

Landscape variable	Abbreviation	Configuration or complexity	Definition
Isolation‐by‐distance	IBD	Configuration	Resistance surface where all pixels assigned value of 1; assumes only distance influences gene flow
Isolation‐by‐barrier	IBB	Configuration	Resistance surface where pixels assigned 1 or 500; assumes distance and open water influence gene flow
Mortality low	MortL	Configuration	Resistance surface where urban pixels assigned probability of dying in urban habitats; assumes six land cover types influence gene flow
Mortality high	MortH	Configuration	Resistance surface where urban pixels assigned probability of dying on roads; assumes six land cover types influence gene flow
Movement low	MoveL	Configuration	Resistance surface where urban pixels assigned probability of moving into urban habitats; assumes six land cover types influence gene flow
Movement high	MoveH	Configuration	Resistance surface where urban pixels assigned probability of moving onto a road; assumes six land cover types influence gene flow
Proportion of forest	PrFor	Complexity	Proportion of forest habitat within each study cell
Patch density	PD	Complexity	The number of forest patches within a study cell
Clumpy	Clumpy	Complexity	Measure of aggregation of forest patches while controlling for PrFor

Landscape configuration variables are composed of resistance surfaces that reflect hypothesized impacts of six land cover types on gene flow between study cells. All resistance surfaces were parameterized according to Rizkalla and Swihart (2012; Table S1). Landscape complexity variables are within study cell landscape metrics that were found to predict either occupancy or abundances in the UWB for chipmunks and white‐footed mice.

For each species, we modeled connectivity employing only a single resistance surface at a time, resulting in six runs per species. Each run in circuitscape assumed that resistance surfaces reflected resistance values instead of conductance values and allowed for nodes to be connected by eight cell neighbors. For each resistance surface input, circuitscape outputs a matrix of landscape resistance distances between all study cells, which serve as our measure of landscape configuration between study cells and explanatory variables in subsequent landscape genetic analyses.

### Landscape genetics: landscape complexity within study cells

While circuitscape allows for quantification of how intervening landscape configuration may impact gene flow, multiple authors have suggested that demographic parameters (e.g., abundance; Nowakowski et al. 2015; density; Busch et al. 2009; effective population size; Weckworth et al. 2013) within study areas also influence gene flow between populations. Abundance, for example, has a powerful influence on dispersal regimes and resultant gene flow in rodents (Cutrera et al. 2005; McEachern et al. 2007; Busch et al. 2009), but many demographic parameters can be difficult to measure at large spatial scales like that of this study. A potential solution to this problem is calculating landscape complexity metrics that are known to be correlated with relevant demographic parameters like abundance. For the UWB, most previous studies involved quantifying how fragmentation impacts population dynamics within forest patches, not across the entire study cell. Abundances within forest patches are known to vary according to a number of complexity variables (Nupp and Swihart 1998; Moore and Swihart 2005; Rizkalla and Swihart 2012), so extrapolating patterns observed in forest patches to an entire study cell is not ideal. Many influences on gene flow are scale dependent (Anderson et al. 2010), so estimating abundances or focusing on metrics important for patch‐based processes likely will not translate to broad‐scale patterns in gene flow. Therefore, we included complexity variables that were relevant for simulated abundances within each study cell instead of previous patch‐based complexity metrics or abundance estimates.

Based on Rizkalla and Swihart (2012), we chose three complexity metrics (proportion of forest, patch density, and Clumpy) that were strongly correlated with simulated abundances within the UWB. The advantage of focusing on complexity metrics that were correlated with simulated abundances is that total abundances were known, so the complexity metrics were both important at the study cell scale and are known to reflect differences in abundance. We calculated proportion of forest, patch density, and Clumpy, a measure of patch aggregation, in fragstats v. 4.2 (McGarigal et al. 2012) based on Moore and Swihart's (2005) reclassified 3 × 3 m rasters of each 23 km^2^ study cell with a 1.6‐km buffer. In total, all statistical models included three landscape complexity metrics as explanatory variables: proportion forest (prFor), patch density (PD), and Clumpy (Table 1).

### Landscape genetics: statistical analysis

We performed two statistical tests to examine the relationship between our measures of landscape configuration and complexity and genetic differentiation (*F*_ST_ and *D*_EST_). Our first analysis used multiple regression on distance matrices (MRDM; Legendre et al. 1994), an extension of Mantel tests that can incorporate multiple pairwise matrices as explanatory variables. MRDM requires that all variables are pairwise distances, so we utilized the average landscape complexity metrics (i.e., prFor, PD, and Clumpy) for each pair of study cells. As a result, we conducted twelve MRDM tests per species, one test per resistance surface for *F*_ST_ or *D*_EST_. Each model included four explanatory variable matrices: one matrix of resistance distances calculated from a resistance surface (IBD, IBB, MortL, MortH, MoveL, or MoveH) and three average landscape complexity metrics. Explanatory variables were eliminated from each MRDM model based on Zuur et al. (2009) where a final model only included variables that were significantly correlated with *F*_ST_ or *D*_EST_. Resultant reduced models, thus, reflect the combination of landscape complexity variables that explain the most variance in genetic differentiation for each resistance (configuration) surface. For the best overall model that combined both configuration and complexity, we selected the model with the highest adjusted *R* ^2^ among the six reduced MRDM models for each genetic distance. Statistical significance of each explanatory variable and *R* ^2^ were calculated via 10,000 permutations of the genetic differentiation matrices within the R package *ecodist* (function “MRM”; Goslee and Urban 2007).

Our second complementary analysis utilized distance‐based redundancy analysis (dbRDA), a multivariate analog to multiple linear regression (Legendre and Legendre 2012). A dbRDA first transforms pairwise response distances (i.e., *F*_ST_ and *D*_EST_) using principal coordinates analysis (PCoA) and extracts all PCoA vectors that have positive eigenvalues. Then, a redundancy analysis is performed with the PCoA vectors as the response variable. Unlike MRDM, dbRDA requires site‐specific explanatory variables, so we transformed the resistance distance matrices into a connectivity index for each study cell using the following equation:

$$S_{i}=\sum \;\;\;\text{exp}\;\;\left(-\alpha d_{ij}\right)$$

where *S*_*i*_ is the connectivity index for study cell *i*,* α* is a scalar correlated with average dispersal distance of the species, and *d* is the resistance distance between sample sites *i* and *j* (Moilanen and Nieminen 2002). Each matrix of resistance distances was transformed into connectivity indices for the 28 study cells, and along with the three complexity metrics (prFor, PD, and Clumpy), served as explanatory variables within twelve dbRDA tests per species (i.e., one for each resistance surface for *F*_ST_ and *D*_EST_). We used the function “capscale” within *vegan* to perform dbRDA tests and function “ordistep” to perform forward selection to eliminate explanatory variables for each resistance surface. Finally, adjusted *R* ^2^ for each reduced dbRDA model was calculated via the function “RsquareAdj” in *vegan*, and like the MRDM analysis, we chose the best reduced dbRDA among the six resistance surfaces based on the highest adjusted *R* ^2^.

While both MRDM and dbRDA provide estimates of model fit (i.e., adjusted *R* ^2^), we sought to calculate measures of variance around each estimate from tested models. Therefore, we bootstrap resampled our dataset for each species 1000 times to calculate means and 95% confidence intervals around each F statistic, regression coefficient, and *R* ^2^ value. All MRDM and dbRDA tests were performed on each bootstrap permutation as described above. This method of resampling allows for further comparison among models, particularly regarding how landscape resistance hypotheses compare to IBD and IBB. Specifically, if the 95% confidence intervals of model fit estimates (i.e., *R* ^2^) of landscape resistance models did not encompass those of IBD and IBB, we considered this strong evidence for landscape effects on gene flow.

## Results

### Population differentiation and diversity

For chipmunks, no consistent deviations in either HWE or LE were observed in any study cell, whereas 2 loci (PO‐26 and Pml02) deviated from HWE in all study cells in white‐footed mice. microchecker suggested that these two loci may contain null alleles (frequency >0.152), so we eliminated PO‐26 and Pml02 from all subsequent analyses. Although genetic diversities were similar between study cells for both species, many study cells exhibited heterozygote deficiencies (Table 2), a potential consequence of population substructure within those cells (i.e., Wahlund effect).

**Table 2:** Genetic diversity metrics for each study cell across 12 and 8 loci for chipmunks and white‐footed mice, respectively

Cell	*N*	Na	*A*_R_	*H*_O_	*H*_E_	*F*_IS_	*F*_IS_ 95% lower	*F*_IS_ 95% upper
Chipmunk								
295	145	11.75	7.04	0.705	0.762	0.075	0.048	0.101
365	19	6.83	5.74	0.722	0.725	0.004	−0.067	0.066
366	131	10.58	7.08	0.715	0.753	0.050	0.025	0.074
400	28	6.92	5.55	0.711	0.715	0.005	−0.061	0.072
459	14	6.17	5.42	0.756	0.710	−0.064	−0.179	0.025
464	157	9.67	6.52	0.702	0.732	0.041	0.017	0.066
580	54	8.50	6.39	0.734	0.748	0.019	−0.024	0.065
613	24	7.17	5.86	0.705	0.696	−0.013	−0.093	0.070
654	67	9.50	6.63	0.730	0.748	0.024	−0.010	0.058
691	19	5.83	5.06	0.675	0.669	−0.009	−0.083	0.065
763	34	7.08	5.59	0.689	0.711	0.031	−0.019	0.079
790	42	8.17	6.14	0.650	0.708	0.082	0.034	0.131
793	36	7.08	5.72	0.690	0.709	0.027	−0.024	0.070
803	28	8.08	6.35	0.738	0.733	−0.006	−0.071	0.058
826	29	8.08	6.17	0.727	0.739	0.017	−0.038	0.071
831	33	7.33	5.97	0.711	0.719	0.012	−0.052	0.078
844	53	7.83	6.00	0.663	0.711	0.068	0.025	0.109
845	19	5.50	4.85	0.675	0.689	0.019	−0.049	0.078
854	53	9.25	6.55	0.698	0.751	0.070	0.031	0.112
856	17	6.33	5.55	0.745	0.677	−0.100	−0.176	−0.038
865	27	7.33	6.14	0.722	0.705	−0.010	−0.057	0.037
869	32	7.25	6.14	0.737	0.743	−0.030	−0.057	0.037
875	25	6.33	5.35	0.718	0.687	−0.044	−0.106	0.012
896	30	6.83	5.54	0.678	0.723	0.063	0.015	0.113
898	23	7.17	5.87	0.712	0.715	0.005	−0.040	0.049
905	32	7.17	5.86	0.691	0.705	0.020	−0.028	0.066
920	40	7.92	6.26	0.740	0.753	0.018	−0.030	0.066
960	17	6.17	5.53	0.692	0.737	0.061	−0.011	0.121
White‐Footed Mouse								
295	22	10.13	8.10	0.676	0.805	0.147	0.052	0.236
365	35	11.75	8.62	0.612	0.812	0.243	0.170	0.314
366	68	16.13	10.54	0.698	0.846	0.166	0.120	0.208
400	35	13.75	9.95	0.741	0.852	0.122	0.062	0.184
459	17	9.75	8.19	0.788	0.813	0.020	−0.068	0.097
464	38	12.50	9.14	0.800	0.842	0.032	−0.020	0.088
580	25	10.75	8.41	0.697	0.817	0.137	0.062	0.208
613	35	11.00	7.78	0.600	0.783	0.225	0.154	0.294
654	90	14.88	9.43	0.654	0.839	0.212	0.172	0.251
691	25	11.63	8.91	0.726	0.812	0.096	0.015	0.176
763	21	10.50	8.53	0.667	0.798	0.142	0.084	0.190
790	20	10.88	8.89	0.809	0.843	0.031	−0.042	0.101
793	20	11.00	8.94	0.823	0.853	0.021	−0.062	0.107
803	30	12.75	9.51	0.774	0.839	0.065	−0.007	0.136
826	38	13.00	9.34	0.726	0.848	0.131	0.063	0.194
831	38	12.38	8.94	0.707	0.825	0.129	0.073	0.188
844	19	10.13	8.39	0.743	0.806	0.072	−0.023	0.149
845	46	12.63	8.96	0.722	0.826	0.117	0.067	0.168
854	20	9.63	7.67	0.733	0.782	0.053	−0.060	0.152
856	23	11.50	8.74	0.703	0.819	0.135	0.051	0.212
865	34	12.25	9.27	0.723	0.830	0.117	0.066	0.172
869	44	13.00	9.25	0.664	0.823	0.183	0.121	0.251
875	35	12.13	9.00	0.728	0.829	0.125	0.068	0.182
896	41	13.88	9.63	0.722	0.830	0.125	0.067	0.183
898	23	11.38	8.97	0.667	0.828	0.198	0.091	0.288
905	40	12.25	8.66	0.692	0.827	0.148	0.087	0.207
920	42	12.25	9.07	0.751	0.832	0.086	0.037	0.133
960	31	12.13	9.16	0.741	0.829	0.102	0.043	0.156

Chipmunks largely adhered to HWE; in that, only two cells had a significantly positive *F*_IS_. In contrast, 21 of 28 study cells exhibited positive *F*_IS_ values in white‐footed mice.

All genetic differentiation metrics for chipmunks were significantly different from zero (all *P* < 0.001) for both *F*_ST_ (range: 0.010–0.125) and *D*_EST_ (range: 0.011–0.309). In contrast, only 77.31% of *F*_ST_ (range: −0.001 to 0.052) and 69.79% of *D*_EST_ (range: 0.001–0.227) values were significantly different from zero in white‐footed mice. For both species, the smallest genetic differentiation values occurred between proximate study cells within the southwestern corner, the most heavily forested study cells, of our study area.

We found that geographic distance impacted genetic differentiation for both species within the UWB, but not the Wabash River (all partial Mantel *r* < 0.125, all *P* > 0.051). Geographic distance had differing impacts on chipmunks and white‐footed mice across the UWB. Chipmunks exhibited strong IBD across the study area (Mantel *r*_FST_ = 0.342, Mantel *r*_DEST_ = 0.377, both *P* < 0.001) and within 25 of 28 study cells (all significant *r* > 0.085, *P* < 0.025: Table S5). Simple Mantel tests were not significant for IBD across the study area for white‐footed mice (Mantel *r*_FST_ = 0.085, Mantel *r*_DEST_ = 0.071, both *P* > 0.225), but 23 of 28 IBD tests within study cells were significant (Mantel *r* > 0.125, *P* < 0.045; Table S5). Despite the differences in IBD test results, both species exhibited significant positive spatial autocorrelations for individuals captured 100 m or less apart (i.e., smallest distance interval within the spatial autocorrelations) in the majority of study cells (27 of 28 in both chipmunks and white‐footed mice; Table S2). Regardless of the size of distance classes within the spatial autocorrelations, results for the smallest distance class remained consistent. Although sample size prohibited distance classes smaller than 50 m, the consistent results across multiple distance classes provide strong evidence for restricted dispersal within study cells.

### Bayesian clustering analysis

**Figure 2:**
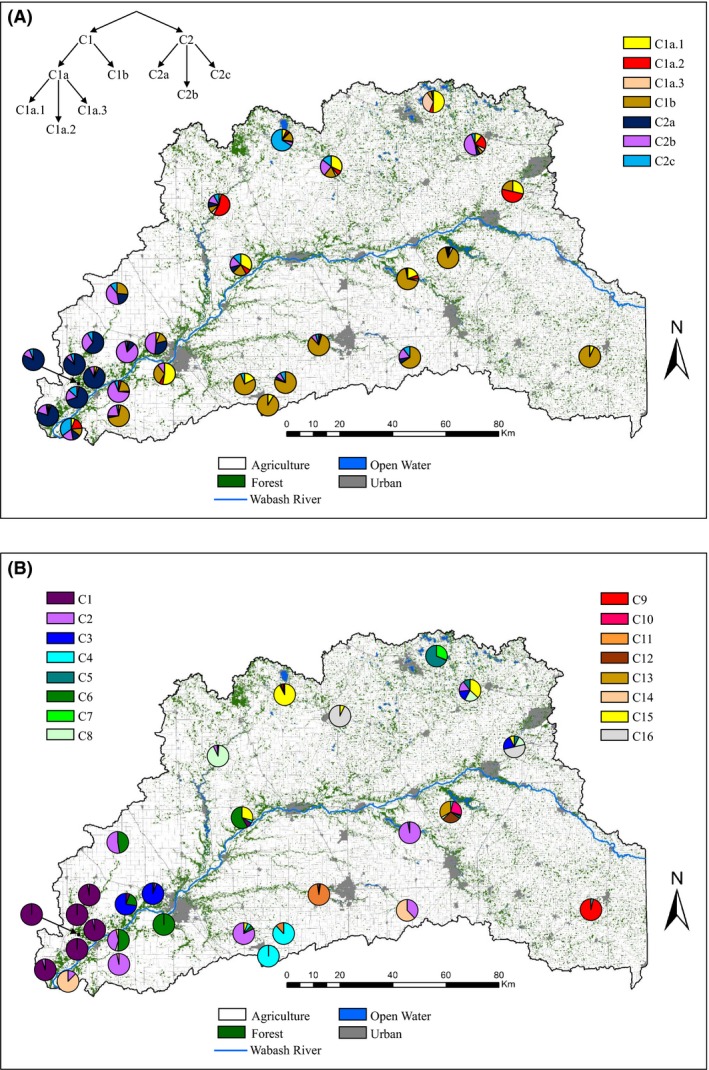
Results of the structure (A) and baps (B) analysis for eastern chipmunks across the UWB. structure revealed complex hierarchical genetic structure (upper left) where after iterative runs, the ending number of putative clusters was seven (C1a.1, C1a.2, Cla.3, Clb, C2a, C2b, C2c). In contrast, baps identified 16 putative clusters for chipmunks. Pie charts represent the proportion of individuals assigned to each cluster within each study cell.

Both Bayesian clustering programs supported considerable genetic structure in chipmunks, but disagreed on the ideal *K*. structure detected evidence for hierarchical structure where the first run's highest Δ*K* occurred at *K *=* *2 regardless if location priors were included (Δ*K* = 57.152 or 82.812 for no priors and location priors, respectively; Fig. S1). The average likelihoods for *K *=* *2 between the no priors (−56303.0) and location priors (−56304.2) runs also supported *K *=* *2. The first major split generally corresponded to an east–west gradient where eastern individuals were highly assigned to the first cluster and western individuals to the second cluster (Fig. 2A). Iterative runs on the first major cluster eventually revealed four additional subclusters, whereas the second major cluster contained three subclusters (Fig. 2A). There was some evidence for further substructure in multiple subclusters (Δ*K* = 15.671–18.752 or 25.062–28.123 for no priors or priors, respectively), but assignments within these clusters were either weak (most *q *=* *0.35–0.75) or clusters only occurred within a single study cell. Based on the strong IBD found within study cells and weak assignments within putative clusters, the further substructure likely reflects a combination of false clusters due to IBD (Frantz et al. 2009) and fine‐scale structure within study cells.

Substructure within study cells clearly influenced the total *K* found in baps, where *K *=* *17 had the modal likelihood (−55434.181). One cluster contained a few individuals (*n* = 4) scattered across the study area, a likely “ghost population” (Corander and Marttinen 2006; Latch et al. 2006), which reduced the optimal *K* to 16. All other clusters contain >20 individuals, so they were considered putative clusters. In general, baps corroborated the major splits detected by structure with additional substructure identified within study cells (Fig. 2B). Similar to the structure results, baps assigned much of the densely sampled southwestern portion of our study area to one cluster with many additional, more isolated clusters detected in the sparsely sampled areas. However, baps can indicate discrete clusters in cases where sampling gaps occur in populations exhibiting IBD (Frantz et al. 2009), which likely explains the differences in the optimal *K* between the Bayesian algorithms.

**Figure 3:**
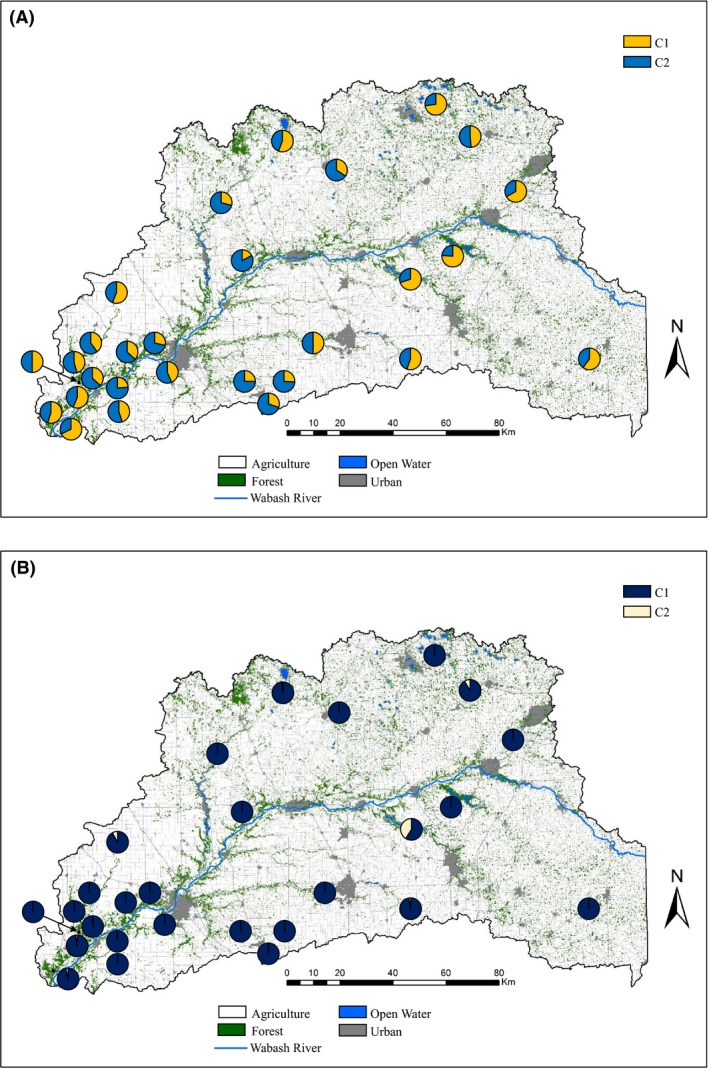
Distribution of two putative clusters found in structure (A) and baps (B) for white‐footed mice across the UWB. Pie charts correspond to the proportion of individuals assigned to each putative cluster (C1 or C2) within a study cell. No apparent genetic differentiation was detected within either the structure or baps analysis for white‐footed mice.

In contrast to chipmunks, neither of the Bayesian programs revealed strong evidence of genetic structure in white‐footed mice across the study area. In structure, the highest Δ*K* occurred at *K *=* *2 for both no priors (4.0444, likelihood = −35425.5) and location priors (8.254, likelihood = −36210.3), but were not distinct from any other *K* (Fig. S2). Furthermore, the majority of *q*‐values ranged from 0.35 to 0.65, and when plotted, the spatial distribution of the clusters had little clarity (Fig. 3A). Therefore, we considered the most likely *K* in structure to be 1. Similarly, the modal likelihood for baps was *K *=* *3 (−36022.152), with the vast majority of individuals assigned to a single cluster (900 of 959 individuals). One of the clusters had only nine individuals and was not considered valid, whereas the second primarily occurred within a single study cell (Fig. 3B). Overall, white‐footed mice showed very little genetic structure with no clear spatial structure as observed in chipmunks.

### Landscape genetics

Landscape configuration between study cells and to a lesser extent complexity within study cells played a role in driving genetic differentiation between study cells for chipmunks, but not in white‐footed mice. In the MRDM analysis for chipmunks, all reduced models (12 total; 6 for each genetic distance) regardless of the resistance surface included resistance distance as a significant explanatory variable (Table S3). Pairwise resistance distances (i.e., measure of landscape configuration between study cells) always were positively correlated with genetic differentiation regardless of genetic distance. No MRDM model included any landscape complexity variables.

The top MRDM model in chipmunks (i.e., the model with the highest adjusted *R* ^2^) among the resistance surfaces differed based on the genetic distance (Table 3). In the six models that had *F*_ST_ as the response variable, the reduced model for the IBD resistance surface (i.e., null model) explained the most variance ($R_{\mathrm{adj}}^{2}$ = 0.071). The 95% confidence intervals around the adjusted *R* ^2^ for the reduced IBD model indicated no models containing alternative resistance surfaces were competing models because the 95% confidence intervals did not overlap. For the six *D*_EST_ reduced models, the reduced model that included the MoveL resistance distances and no complexity variables explained the most variance ($R_{\mathrm{adj}}^{2}$ = 0.106; Table 3). No other models were considered top models because their 95% confidence intervals around adjusted *R* ^2^ did not overlap those of the MoveL resistance surface.

**Table 3:** Reduced model statistics for multiple regression on distance matrices (MRDM) that quantified the relationship between genetic differentiation (*F*_ST_ and *D*_EST_) and landscape variables (i.e., configuration and complexity) in chipmunks and white‐footed mice

Resistance surface	*F*_ST_					*D*_EST_				
	Avg F	$R_{\mathrm{adj}}^{2}$	95% upper	95% lower	*P* value	Avg *F*	$R_{\mathrm{adj}}^{2}$	95% upper	95% lower	*P* value
Chipmunk										
IBD	**26.754**	**0.071**	**0.072**	**0.070**	**0.006**	40.301	0.103	0.104	0.102	0.001
IBB	20.610	0.055	0.056	0.054	0.017	35.770	0.093	0.094	0.092	0.002
MortL	22.499	0.060	0.061	0.060	0.022	38.583	0.099	0.100	0.098	0.002
MortH	22.207	0.059	0.060	0.058	0.023	36.781	0.095	0.096	0.094	0.003
MoveL	23.317	0.062	0.063	0.061	0.024	**41.061**	**0.106**	**0.105**	**0.104**	**0.001**
MoveH	24.184	0.065	0.066	0.064	0.009	38.759	0.101	0.101	0.100	0.003
White‐footed Mouse										
IBD	2.842	0.023	0.024	0.022	0.683	2.535	0.018	0.020	0.017	0.674
IBB	2.937	0.028	0.029	0.027	0.617	2.727	0.021	0.022	0.020	0.656
MortL	3.152	0.030	0.031	0.029	0.623	3.122	0.029	0.030	0.028	0.625
MortH	3.218	0.033	0.034	0.032	0.618	3.388	0.031	0.032	0.031	0.622
MoveL	3.023	0.030	0.032	0.029	0.621	3.292	0.030	0.031	0.029	0.666
MoveH	**3.329**	**0.037**	**0.038**	**0.036**	**0.608**	**3.417**	**0.032**	**0.033**	**0.031**	**0.611**

The reduced model that explained the most variance among the six resistance surfaces (IBD, IBB, MortL, MortH, MoveL, MoveH) is bolded. For *F*_ST_, all reduced models only contained resistance distances and Clumpy as a significant variables, whereas models with *D*_EST_ contained resistance distances only (Table S3). Average *F* statistics (Avg *F*), *P* values (*P* value), adjusted *R* ^2^, and 95% confidence intervals around *R* ^2^ are provided for each reduced model and were calculated based on 1000 bootstrap iterations.

The dbRDA analysis in chipmunks was complementary to the results of the MRDM analysis because it found that configuration (connectivity indices) was important in explaining genetic differentiation (Table S4). Results from the dbRDA models also suggested that Clumpy affected genetic differentiation unlike the MRDM analyses. All reduced dbRDA models for *F*_ST_ and *D*_EST_ contained both connectivity indices derived from resistance distances and Clumpy. Likewise, the top reduced models among the six resistance surfaces for both measures of genetic distance (12 total reduced models) incorporated connectivity indices calculated from the MortH resistance surface and Clumpy (*F*_ST_: $R_{\mathrm{adj}}^{2}$ = 0.156, *D*_EST_: $R_{\mathrm{adj}}^{2}$ = 0.156; Table 4). Calculated 95% confidence intervals around each top model's adjusted *R* ^2^ did not overlap any competing models, and thus, the top models explained more variance than both our null resistance surfaces (IBB and IBD). Unlike the MRDM analysis, dbRDA found that resistance surfaces with highly resistant urban habitat explained genetic variation better than those with weak resistances for urban habitat This counterintuitive results indicate that MRDM, an extension of Mantel tests, may suffer from reduced power to detect landscape genetic patterns (Legendre and Fortin 2010). By collapsing resistance distances into a single connectivity metric for dbRDA, we obtained stronger evidence for landscape effects on gene flow. Therefore, if MRDM failed to detect Clumpy due to reduced power, both landscape configuration and complexity appear to influence gene flow in chipmunks based on dbRDA.

**Table 4:** Results from the distance‐based redundancy analysis (dbRDA) for eastern chipmunks relating landscape variables and connectivity indices to two measures of genetic differentiation (*F*_ST_ and *D*_EST_)

Resistance surface	*F*_ST_					*D*_EST_				
	Avg *F*	$R_{\mathrm{adj}}^{2}$	95% upper	95% lower	*P* value	Avg *F*	$R_{\mathrm{adj}}^{2}$	95% upper	95% lower	*P* value
IBD	2.870	0.119	0.120	0.117	0.009	2.771	0.116	0.117	0.115	0.010
IBB	2.981	0.128	0.130	0.127	0.008	2.985	0.129	0.130	0.128	0.008
MortL	3.366	0.154	0.155	0.152	0.006	3.348	0.153	0.154	0.152	0.006
MortH	**3.404**	**0.156**	**0.157**	**0.155**	**0.006**	**3.414**	**0.156**	**0.158**	**0.155**	**0.005**
MoveL	3.123	0.140	0.142	0.139	0.006	3.056	0.136	0.138	0.135	0.007
MoveH	3.228	0.146	0.148	0.144	0.006	3.200	0.145	0.146	0.143	0.006

Forward selection for all dbRDA tests in white‐footed mice always chose the null model. Comparisons between reduced models for each resistance surface (IBD, IBB, MortL, MortH, MoveL, and MoveH) revealed that the model that incorporated MortH and Clumpy of forest habitats explained the most variance ($R_{\mathrm{adj}}^{2}$) among the reduced models (bolded, parameter estimates are provided in Table S4). Average *F* statistics (Avg *F*), adjusted *R* ^2^ with 95% confidence intervals, and average *P* values (*P* value) are provided for each reduced model and were calculated based on 1000 bootstrap iterations.

Results for white‐footed mice, in contrast, supported the evidence for limited genetic structure detected by the Bayesian clustering analysis. No configuration or complexity variables were significant for any resistance surface in the MRDM analyses. Similarly, forward selection in dbRDA always selected the null model across the 1000 bootstrap permutations for each resistance surface. Overall, it appears that landscape configuration and complexity had little impact on gene flow in white‐footed mice.

## Discussion

Despite the ubiquity of chipmunks and white‐footed mice across the highly fragmented UWB, these species exhibited dramatically different patterns of genetic structure across the landscape. Chipmunks exhibited strong IBD, hierarchical genetic clustering, and patterns of genetic differentiation that were correlated with both resistance distances and patch aggregation (Clumpy). White‐footed mice, in contrast, had an overall lack of genetic structure with no signal of IBD, no discrete genetic clusters, and no evidence of landscape effects on genetic structure across the >20,000 km^2^ UWB. Taken together, it appears that chipmunks’ greater dependence on forest cover explains the differences in gene flow observed between chipmunks and white‐footed mice. Thus, niche specialization on forests appears to be a more powerful influence on our focal species’ gene flow and corresponding sensitivities to fragmentation than body size within the UWB.

### Gene flow of eastern chipmunks and white‐footed mice

Genetic differentiation in both chipmunks and white‐footed mice was strongly correlated with geographic distance within study cells. The simple Mantel tests and spatial autocorrelations suggested restricted dispersal within the majority of study cells, particularly with individuals separated by less than 100 m (i.e., within a trapping grid or patch). For chipmunks, fine‐scale genetic differentiation in our study area is consistent with other studies that have documented short dispersal distances in both sexes (<200 m; Loew 2000; Messier et al. 2012). In general, recorded dispersal distances in white‐footed mice are shorter than those of chipmunks (<100 m; Jacquot and Vessey 1995), but spatial autocorrelations revealed evidence for restricted dispersal in both species. Therefore, the larger body size of chipmunks may not translate to greater gene flow than white‐footed mice within this agricultural ecosystem.

Despite the similar levels of fine‐scale genetic structure within study cells, we detected dramatically different levels of putative gene flow and genetic differentiation in eastern chipmunks and white‐footed mice across the UWB. Eastern chipmunks exhibited strong IBD and formed a number of discrete genetic clusters, whereas white‐footed mice formed a large, panmictic population. The complex, hierarchical structure of eastern chipmunks was primarily driven by IBD and to a lesser extent by landscape heterogeneity across the UWB. structure revealed at least three different layers of clustering for chipmunks where the major split revealed an east–west gradient, likely a result of IBD. Another distinct pattern was the high connectivity (i.e., most study cells were assigned to a single cluster) within the southwestern portion of the study area along the Wabash River. Forests were concentrated along rivers, so the presence of relatively continuous habitat within the southwestern area of the study area likely facilitated chipmunk gene flow between study cells. In contrast, many more clusters were found in the eastern portion of the study area than the southwestern area, but it is difficult to determine whether genetic differentiation was due to the greater proportion of agricultural lands between study cells or simply the large gaps between study cells that had suitable numbers of chipmunks. Sampling along a genetic gradient (i.e., IBD) in combination with low levels of genetic differentiation can create false clusters within Bayesian programs (e.g., Latch et al. 2006; Frantz et al. 2009; Schwartz and McKelvey 2009), so the diffuse sampling and large tracts of unsuitable habitat between study cells both could have contributed the high number of clusters within the eastern portion of our study area. Regardless of sampling, the high connectivity observed within the southwestern area of the study area and high differentiation within the less forested regions provide evidence for forest being an important driver of gene flow within eastern chipmunks.

Further evidence for forest driving gene flow in chipmunks was found in the landscape genetics analysis, although variation explained by the best models was low. In all analyses but *F*_ST_ MRDM tests (18 of 24), MRDM and dbRDA suggested that landscape configuration metrics (i.e., resistance surfaces) explained more variance than IBD. Combined with the Bayesian clustering results and previous fine‐scale studies (Anderson et al. 2015), our landscape genetic analysis suggests that forests promote gene flow between study cells for chipmunks. Complexity of forested habitat within study cells may also play a role in explaining patterns of genetic differentiation in chipmunks. Similar to resistance surfaces, dbRDA found aggregation of forests within study cells to impact gene flow where study cells with similar Clumpy metrics had lower genetic differentiation. This pattern was largely driven by the study cells with highly aggregated forest patches (i.e., southwestern study cells) that experienced high levels of gene flow according to the Bayesian clustering programs. Less fragmented patches (i.e., greater clumpiness index) of suitable habitat have been recorded to facilitate gene flow in both empirical (Kelly et al. 2014) and simulated (Kierepka and Latch 2015) studies of fragmentation, so it appears that chipmunks experience higher gene flow in less fragmented forested habitats.

Although we found strong evidence for forests as a facilitator of gene flow, MRDM and dbRDA disagreed on how urban habitat impacts gene flow in chipmunks. MRDM implied that urban habitat was not a strong barrier to gene flow (i.e., MortL had the lowest resistance for urban), whereas the most variance in dbRDA was explained with MoveH, the model with the highest resistance for urban. Several possibilities could explain this discrepancy. First, only dbRDA controls for the effects of IBD, so MDRM may have not had the power to differentiate landscape effects from IBD. Urban habitats, principally large roads, have been suggested to be barriers to chipmunks (Oxley et al. 1974; Ford and Fahrig 2008; McGregor et al. 2008), which agrees with the dbRDA results. The MRDM results, in contrast, suggest that the reduced movement across roads may not translate to genetic differentiation, perhaps due to a time lag (Landguth et al. 2010) or high effective population sizes (Gauffre et al. 2008). Roads did not separate genetic clusters in a previous fine‐scale analysis of chipmunks (Anderson et al. 2015), but Hennessy (2012) found that large interstates within Indiana form substantial genetic barriers for both chipmunks and white‐footed mice. Therefore, the roads in the UWB may not be large enough to cause observable genetic differentiation within our focal species. Alternatively, studies that detect genetic differentiation often involve targeted sampling along focal roads (e.g., Riley et al. 2006; Frantz et al. 2012; Hennessy 2012; Marsh et al. 2012), so more directed sampling could elucidate whether roads or urban habitat inhibit chipmunk gene flow within the UWB.

Unlike chipmunks, geographic distance and forested habitat had little impact on genetic structure in white‐footed mice across the UWB. Neither Bayesian program found evidence of genetic substructure, and all landscape genetic analyses indicated that one of our null models (e.g., IBD) fit the data best. Despite white‐footed mice being a generalist, the overall lack of genetic structure across the UWB was surprising because they possess several ecological and life‐history traits that would favor the formation of genetic structure. Decreased maximum movement by white‐footed mice within agricultural fields as compared to chipmunks (Rizkalla and Swihart 2007) and limited perceptual range of white‐footed mice in agricultural fields (Zollner and Lima 1997) both suggest that fragmentation within the UWB should lead to genetic differentiation much like that observed for urban populations (Munshi‐South 2012). Despite such predictions, however, the lack of genetic structure we observed in this species corroborates the observations of previous investigations (Mossman and Waser 2001). Thus, while seemingly unlikely, it is quite possible that white‐footed mice traverse agricultural fields more successfully than their size and mobility would predict (Cummings and Vessey 1994), especially if corridors such as fencerows are present.

In addition, white‐footed mice can persist at high abundances in agricultural ecosystems (Cummings and Vessey 1994), and high population abundances and occupancy rates have been documented for this species within the UWB (Nupp and Swihart 1998; Moore and Swihart 2005). Given their high abundances, occupancy rates, and genetic diversity within study cells, white‐footed mice likely maintain high effective population sizes across the UWB, which in turn resists genetic drift and subsequent spatial differentiation (e.g., Gauffre et al. 2008; Rico et al. 2009). Therefore, despite apparent limitations in movement, the high effective population sizes of white‐footed mice may counteract the isolating factors of fragmentation, resulting in the panmictic population structure observed within our dataset.

### Agreement between genetic and demographic effects of fragmentation

While our broad prediction that ecological specialization would enhance genetic structure in chipmunks inhabiting the UWB agroecosystem was well supported, congruence between our study and previous studies of how habitat alteration impacts rodents within the UWB was mixed. Chipmunk gene flow generally agreed with hypotheses for landscape configuration derived from Rizkalla and Swihart (2012), which provides further evidence that chipmunk dependence on forest habitat drives both within study cell parameters (i.e., abundance; Nupp and Swihart 1998; Rizkalla and Swihart 2012; occupancy; Moore and Swihart 2005) and gene flow (Anderson et al. 2015). In contrast, most landscape complexity metrics were not correlated with genetic differentiation in either species. The latter result is consistent with Swihart et al. (2006), who as part of a multispecies analysis found that residual variation in cell‐level occupancy of chipmunks and white‐footed mice was not explained by landscape complexity metrics after accounting for variation due to niche specialization, proximity to range boundary, and phylogeny. Nonetheless, the lack of relationship between landscape complexity and genetic differentiation was surprising given that the same complexity metrics predicted abundance in the UWB (Rizkalla and Swihart 2012), and dispersal in eastern chipmunks and white‐footed mice is related to environmental conditions in other populations (Morris and Diffendorfer 2004; Messier et al. 2012). A number of factors can contribute to incongruencies between field and genetic‐based studies (e.g., Gauffre et al. 2008; Spear et al. 2010; Wasserman et al. 2010; Mateo‐Sánchez et al. 2015), but we hypothesize that a combination of large effective population sizes, particularly in white‐footed mice, and scale masked the influence of landscape complexity on gene flow.

Effects of fragmentation on populations can be measured in multiple ways including local abundance, occupancy, and genetics, but studies indicate the strength of correlations between these effects and landscape heterogeneity varies both across space and over time (e.g., Anderson et al. 2010; Jackson and Fahrig 2014). Effects of landscape heterogeneity on abundance or occupancy may be strong within study cells (Moore and Swihart 2005; Rizkalla and Swihart 2012), but genetic effects of fragmentation (i.e., losses in genetic diversity; Jackson and Fahrig 2014), appear to be most readily detected at broad spatial scales. This mismatch in spatial scales, therefore, may result in weak correlations between landscape complexity and gene flow across the UWB. Furthermore, genetic variation reflects gene flow across multiple generations, so any fluctuations in dispersal regimes (e.g., Messier et al. 2012) can obscure how landscape heterogeneity impacts overall gene flow. Based on our results and the difficulty in controlling the effects of spatial and temporal effects on gene flow, combining genetics and field‐based studies at multiple scales will give the most complete understanding of how fragmentation impacts focal populations.

## Conclusions

Overall, our results highlight that habitat alteration has complex impacts on even common species such as eastern chipmunks and white‐footed mice. Similar to previous comparative studies (e.g., Brouat et al. 2003; DiLeo et al. 2010; Shanahan et al. 2011; Engler et al. 2014), we found that fragmentation more strongly impacted gene flow in the more specialized species (i.e., chipmunks) despite their larger body size. Chipmunk gene flow was related to both landscape configuration between study cells and landscape complexity (aggregation of forested habitat) within study cells, whereas the generalist white‐footed mouse formed a large, panmictic population across this complex agricultural ecosystem. Based on our results, we caution equating larger body size to higher gene flow, especially in fragmented landscapes where realized dispersal distances will depend on the distribution of suitable habitat. In addition, the predictors of abundance we considered were poor predictors of gene flow. Only one of the three complexity metrics associated with simulated abundance was correlated with genetic differentiation in either species, so predicting how gene flow occurs in fragmented landscapes can be difficult even in well‐characterized landscapes such as the UWB. Based on the incongruence between our genetic study and previous field‐based studies within the UWB, detection of the negative consequences of fragmentation appears to greatly depend on scale, so focusing on a single spatial or temporal scale may miss critical demographic and genetic processes important for persistence in fragmented landscapes. Therefore, effective management programs should consider multiple lines of evidence (e.g., occupancy, abundance, and gene flow) that vary according to scale to gain a more complete understanding of how species respond to habitat fragmentation.

## Conflict of Interest

None declared.

## Supporting information


**Table S1.** Resistance values of each resistance surface for chipmunks and white footed mice.
**Table S2.** Results from within study cell analyses of Mantel tests and spatial autocorrelations in chipmunks and white‐footed mice.
**Table S3.** Parameter estimates for reduced models for each resistance surface (IBD, IBB, MortL, MortH, MoveL, MoveH) that quantified the relationship between landscape variables (i.e., landscape configuration and complexity) and genetic distance (*F*_ST_ and *D*_EST_) in eastern chipmunks.
**Table S4.** Parameter estimates for significant landscape variables (configuration and complexity) within the reduced dbRDA models for eastern chipmunks.
**Figure S1.** Results of the structure analysis of *K* = 1 to 20 for all sampled chipmunks (*n* = 1229) across the UWB.
**Figure S2.** Results of the Structure analysis for all white‐footed mice (*n* = 959) across the UWB.
