# Supplementary material for: Evaluating the influence of life‐history characteristics on genetic structure: a comparison of small mammals inhabiting complex agricultural landscapes
Source: Ecol Evol. 2016 Aug 18;6(17):6376–96. doi: 10.1002/ece3.2269 (PMC5016657; doi:10.1002/ece3.2269)
Supplement: Supplementary file 1 — Table S1. Resistance values of each resistance surface for chipmunks and white footed mice. Table S2. Results from within study cell analyses of Mantel tests and spatial autocorrelations in chipmunks and white‐footed mice. Table S3. Parameter estimates for reduced models for each resistance surface (IBD, IBB, MortL, MortH, MoveL, MoveH) that quantified the relationship between landscape variables (i.e., landscape configuration and complexity) and genetic distance (F ST and D EST) in eastern chipmunks. Table S4. Parameter estimates for significant landscape variables (configuration and complexity) within the reduced dbRDA models for eastern chipmunks. Figure S1. Results of the structure analysis of K = 1 to 20 for all sampled chipmunks (n = 1229) across the UWB. Figure S2. Results of the Structure analysis for all white‐footed mice (n = 959) across the UWB. [file ECE3-6-6376-s001.docx]

Supplemental Tables and Figures

Table S1: Resistance values of each resistance surface for chipmunks and white footed mice. Resistance values were derived from the probability of mortality (MortL and MortH) or movement (MoveL and MoveH) through each land cover type based on Rizkalla and Swihart (2012). These probabilities were converted to resistance values where the lowest resistance (1) was always assigned to forest and all other resistances were scaled based on their relationship to forest within Rizkalla and Swihart (2012). The category urban was given a resistance based on a low (L) or high (H) probability of mortality or movement. Isolation-by-distance (IBD) and isolation-by-barrier (IBB) models did not differentiate between land cover types and served as null hypotheses within the landscape genetic analyses.

|  | IBD | IBB | MortL | MortH | MoveL | MoveH |
| --- | --- | --- | --- | --- | --- | --- |
| *Chipmunk* |  |  |  |  |  |  |
| Agriculture | 1 | 1 | 2 | 2 | 3 | 3 |
| Forest | 1 | 1 | 1 | 1 | 1 | 1 |
| Grassland | 1 | 1 | 2 | 2 | 2 | 2 |
| Open Water | 1 | 500 | 500 | 500 | 500 | 500 |
| Other | 1 | 1 | 5 | 5 | 5 | 5 |
| Urban | 1 | 1 | 5 | 30 | 3 | 17 |
| Wetland | 1 | 1 | 5 | 5 | 5 | 5 |
| *White footed Mouse* | |  |  |  |  |  |
| Agriculture | 1 | 1 | 2 | 2 | 2 | 2 |
| Forest | 1 | 1 | 1 | 1 | 1 | 1 |
| Grassland | 1 | 1 | 3 | 3 | 2 | 2 |
| Open Water | 1 | 500 | 500 | 500 | 500 | 500 |
| Other | 1 | 1 | 5 | 5 | 5 | 5 |
| Urban | 1 | 1 | 5 | 60 | 2 | 13 |
| Wetland | 1 | 1 | 5 | 5 | 5 | 5 |

Table S2: Results from within study cell analyses of Mantel tests and spatial autocorrelations in chipmunks and white-footed mice. Both species exhibited strong evidence of restricted dispersal within study cells with significant Mantel correlation coefficients (Mantel *r*) in the majority of study cells (25/28 and 23/28 study cells for chipmunks and white-footed mice respectively). Similarly, significantly positive spatial autocorrelation coefficients (SA *r*) were found in 28/29 study cells for both species. The spatial autocorrelation coefficient for each study cell is reported for 0-100 m distance class only as no consistent patterns were observed beyond the smallest distance interval. Coefficients are provided for each study cell with the p-value in parentheses. All p-values were calculated after 10,000 permutations.

|  |  | Chipmunks | |  | White-footed Mice | |
| --- | --- | --- | --- | --- | --- | --- |
| Cell |  | Mantel r | SA *r* |  | Mantel *r* | SA *r* |
| 295 |  | 0.279 (0.001) | 0.153 (0.001) |  | 0.267 (0.002) | 0.172 (0.001) |
| 365 |  | 0.085 (0.186) | 0.132 (0.001) |  | 0.198 (0.003) | 0.143 (0.001) |
| 366 |  | 0.215 (0.001) | 0.111 (0.001) |  | 0.197 (0.001) | 0.114 (0.001) |
| 400 |  | 0.216 (0.040) | 0.084 (0.001) |  | 0.199 (0.010) | 0.167 (0.001) |
| 459 |  | 0.364 (0.025) | 0.045 (0.016) |  | 0.344 (0.010) | 0.146 (0.001) |
| 464 |  | 0.183 (0.001) | 0.117 (0.001) |  | 0.397 (0.001) | 0.104 (0.001) |
| 580 |  | 0.547 (0.001) | 0.211 (0.001) |  | na^a^ | na |
| 613 |  | 0.407 (0.001) | 0.148 (0.001) |  | 0.205 (0.005) | 0.075 (0.001) |
| 654 |  | 0.239 (0.001) | 0.151 (0.001) |  | 0.247 (0.001) | 0.109 (0.001) |
| 691 |  | 0.293 (0.001) | 0.106 (0.002) |  | 0.135 (0.078) | 0.137 (0.001) |
| 763 |  | 0.393 (0.001) | 0.215 (0.001) |  | 0.356 (0.001) | 0.054 (0.001) |
| 790 |  | 0.169 (0.001) | 0.165 (0.001) |  | 0.495 (0.002) | 0.015 (0.001) |
| 793 |  | 0.102 (0.105) | 0.083 (0.001) |  | 0.069 (0.322) | 0.093 (0.001) |
| 803 |  | 0.260 (0.003) | 0.145 (0.001) |  | 0.246 (0.030) | 0.083 (0.001) |
| 826 |  | 0.279 (0.001) | 0.271 (0.001) |  | 0.167 (0.001) | 0.141 (0.001) |
| 831 |  | 0.176 (0.100) | 0.132 (0.001) |  | 0.213 (0.001) | 0.148 (0.001) |
| 844 |  | 0.261 (0.001) | 0.130 (0.001) |  | 0.149 (0.071) | 0.172 (0.001) |
| 845 |  | 0.174 (0.022) | 0.001 (0.704) |  | 0.128 (0.009) | 0.113 (0.001) |
| 854 |  | 0.326 (0.001) | 0.185 (0.001) |  | 0.368 (0.001) | 0.064 (0.001) |
| 856 |  | 0.445 (0.002) | 0.047 (0.001) |  | 0.125 (0.045) | 0.050 (0.002) |
| 865 |  | 0.245 (0.001) | 0.062 (0.001) |  | 0.223 (0.006) | 0.101 (0.001) |
| 869 |  | 0.346 (0.001) | 0.126 (0.001) |  | 0.161 (0.038) | 0.089 (0.001) |
| 875 |  | 0.400 (0.001) | 0.321 (0.001) |  | -0.025 (0.361) | 0.160 (0.001) |
| 896 |  | 0.232 (0.001) | 0.172 (0.001) |  | 0.021 (0.370) | 0.098 (0.001) |
| 898 |  | 0.345 (0.001) | 0.232 (0.001) |  | 0.210 (0.008) | 0.104 (0.001) |
| 905 |  | 0.257 (0.001) | 0.074 (0.001) |  | 0.184 (0.001) | 0.082 (0.001) |
| 920 |  | 0.215 (0.002) | 0.097 (0.001) |  | 0.264 (0.002) | 0.139 (0.001) |
| 960 |  | 0.375 (0.001) | 0.017 (0.005) |  | 0.363 (0.001) | 0.174 (0.001) |

^a^All individuals were captured within the same trapping grid

Table S3: Parameter estimates for reduced models for each resistance surface (IBD, IBB, MortL, MortH, MoveL, MoveH) that quantified the relationship between landscape variables (i.e., landscape configuration and complexity) and genetic distance (F_ST_ and D_EST_) in eastern chipmunks. All reduced models for both F_ST_ and D_EST_ contained the resistance distance (Resist) derived from each resistance surface. No models for white-footed mice were significant. Parameter estimates (Avg Est), standard errors (SE), 95% confidence intervals (95% Upper and Lower) and p-values for each parameter (p-value) were calculated via 1000 bootstrap iterations.

|  |  | F_ST_ | | | | | | |  | D_EST_ | | | | |
| --- | --- | --- | --- | --- | --- | --- | --- | --- | --- | --- | --- | --- | --- | --- |
| Resistance Surface | Landscape Variable | Avg Est | | | SE | 95% Upper | 95% Lower | p-value |  | Avg Est | SE | 95% Upper | 95% Lower | p-value |
| IBD | Intercept | | 0.0396 | 0.0000 | | 0.0398 | 0.0394 | 0.998 |  | 0.0595 | 0.0002 | 0.0599 | 0.0590 | 0.999 |
|  | Resist | | 0.0172 | 0.0000 | | 0.0173 | 0.0170 | 0.006 |  | 0.0517 | 0.0052 | 0.0520 | 0.0514 | 0.001 |
| IBB | Intercept | | 0.0429 | 0.0000 | | 0.0430 | 0.0427 | 0.992 |  | 0.0644 | 0.0002 | 0.0649 | 0.0640 | 0.999 |
|  | Resist | | 0.0143 | 0.0000 | | 0.0144 | 0.0142 | 0.017 |  | 0.0463 | 0.0001 | 0.0466 | 0.0460 | 0.002 |
| MortL | Intercept | | 0.0429 | 0.0000 | | 0.0430 | 0.0427 | 0.991 |  | 0.0716 | 0.0024 | 0.0720 | 0.0712 | 0.999 |
|  | Resist | | 0.0143 | 0.0000 | | 0.0144 | 0.0142 | 0.017 |  | 0.0223 | 0.0000 | 0.0224 | 0.0221 | 0.002 |
| MortH | Intercept | | 0.0459 | 0.0000 | | 0.0461 | 0.0458 | 0.989 |  | 0.0757 | 0.0001 | 0.0760 | 0.0753 | 0.999 |
|  | Resist | | 0.0061 | 0.0000 | | 0.0062 | 0.0060 | 0.023 |  | 0.0193 | 0.0000 | 0.0195 | 0.0193 | 0.003 |
| MoveL | Intercept | | 0.0457 | 0.0000 | | 0.0458 | 0.0455 | 0.987 |  | 0.0737 | 0.0002 | 0.0741 | 0.0733 | 0.999 |
|  | Resist | | 0.0051 | 0.0000 | | 0.0052 | 0.0050 | 0.026 |  | 0.0165 | 0.0000 | 0.0166 | 0.0164 | 0.001 |
| MoveH | Intercept | | 0.0461 | 0.0000 | | 0.0462 | 0.0459 | 0.989 |  | 0.0774 | 0.0001 | 0.0771 | 0.0765 | 0.999 |
|  | Resist | | 0.0045 | 0.0000 | | 0.0046 | 0.0044 | 0.022 |  | 0.0143 | 0.0000 | 0.0144 | 0.0141 | 0.003 |

Table S4: Parameter estimates for significant landscape variables (configuration and complexity) within the reduced dbRDA models for eastern chipmunks. For each resistance surface (IBD, IBB, MortL, MortH, MoveL, and MoveH), reduced models for both genetic distances (F_ST_ and D_EST_) included connectivity indices calculated from the resistance surface (Connect) and Clumpy. Average F values (Avg F), standard errors (SE), 95% confidence interval boundaries (95% Upper and Lower), and average p-values (p-value) for each landscape variable was calculated via 1000 bootstrap iterations.

|  |  | | F_ST_ | | | | | | |  | | D_EST_ | | | | | |
| --- | --- | --- | --- | --- | --- | --- | --- | --- | --- | --- | --- | --- | --- | --- | --- | --- | --- |
| Resistance Surface | Landscape Variable | | Avg F | | SE | 95% Upper | 95% Lower | p-value |  | | Avg F | | SE | 95% Upper | 95% Lower | p-value |  |
| IBD | Connect | 2.092 | | 0.012 | | 2.127 | 2.058 | 0.085 |  | | 2.214 | | 0.011 | 2.247 | 2.182 | 0.075 |  |
|  | Clumpy | 3.393 | | 0.018 | | 3.446 | 3.341 | 0.021 |  | | 3.155 | | 0.014 | 3.196 | 3.115 | 0.022 |  |
| IBB | Connect | 2.336 | | 0.010 | | 2.366 | 2.305 | 0.057 |  | | 2.569 | | 0.011 | 2.603 | 2.535 | 0.047 |  |
|  | Clumpy | 3.506 | | 0.014 | | 3.548 | 3.465 | 0.015 |  | | 3.285 | | 0.013 | 3.322 | 3.248 | 0.019 |  |
| MortL | Connect | 3.140 | | 0.013 | | 3.177 | 3.103 | 0.024 |  | | 3.423 | | 0.014 | 3.464 | 3.381 | 0.019 |  |
|  | Clumpy | 3.592 | | 0.016 | | 3.638 | 3.546 | 0.015 |  | | 3.273 | | 0.014 | 3.314 | 3.232 | 0.023 |  |
| MortH | Connect | 3.382 | | 0.015 | | 3.427 | 3.338 | 0.021 |  | | 3.803 | | 0.014 | 3.846 | 3.760 | 0.015 |  |
|  | Clumpy | 3.425 | | 0.017 | | 3.474 | 3.376 | 0.020 |  | | 3.026 | | 0.013 | 3.065 | 2.986 | 0.025 |  |
| MoveL | Connect | 2.753 | | 0.013 | | 2.791 | 2.715 | 0.036 |  | | 2.943 | | 0.013 | 2.982 | 2.904 | 0.022 |  |
|  | Clumpy | 3.493 | | 0.016 | | 3.540 | 3.446 | 0.015 |  | | 3.168 | | 0.014 | 3.210 | 3.126 | 0.025 |  |
| MoveH | Connect | 3.086 | | 0.017 | | 3.136 | 3.035 | 0.022 |  | | 3.437 | | 0.015 | 3.481 | 3.392 | 0.019 |  |
|  | Clumpy | 3.370 | | 0.018 | | 3.423 | 3.318 | 0.020 |  | | 2.962 | | 0.016 | 3.009 | 2.916 | 0.028 |  |


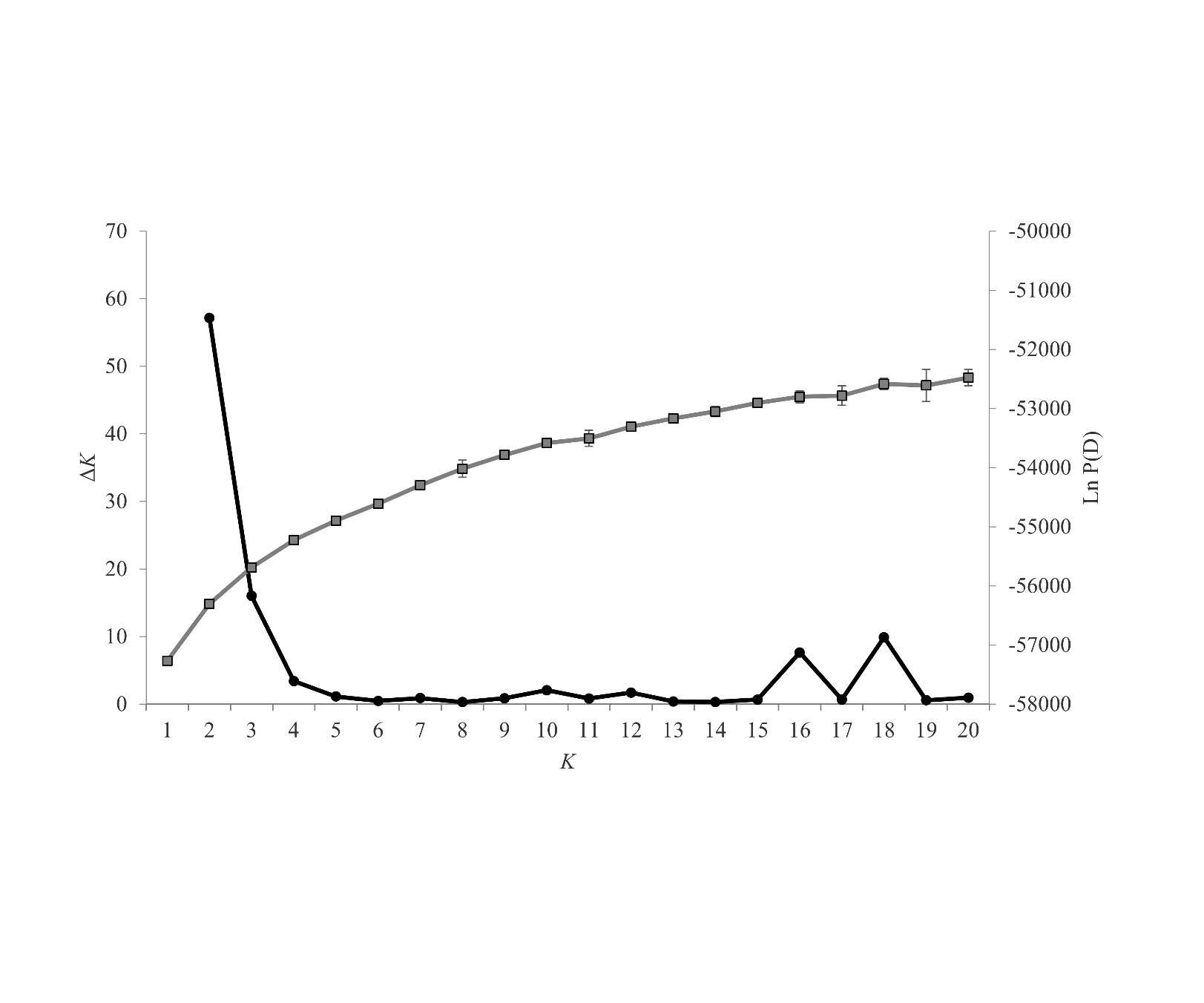


Figure S1. Results of the Structure analysis of *K* = 1 to 20 for all sampled chipmunks (n = 1229) across the UWB. Only the results from the analysis of no priors is shown, but the results were concordant between no priors and location priors. Δ*K* exhibited a large peak at *K* = 2 (black circles), which generally split chipmunks along an east-west gradient (Figure 2). The likelihoods (Ln P(D); grey squares) are depicted for each *K* across 10 iterations per *K*. Error bars correspond to standard deviations around each likelihood.


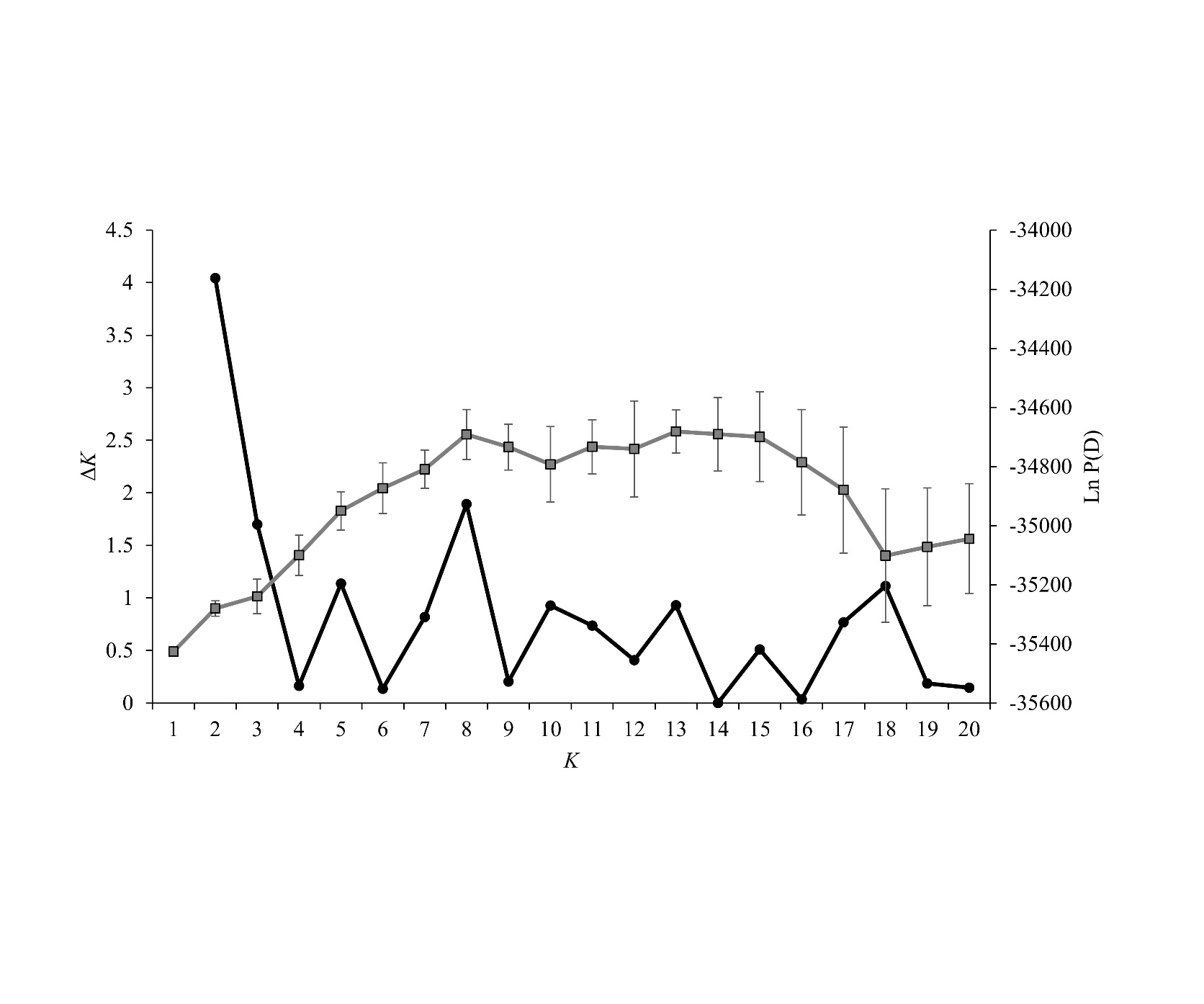


Figure S2. Results of the Structure analysis for all white-footed mice (n = 959) across the UWB. The highest Δ*K* (black circles) occurred at K = 2 regardless if priors were included (Δ*K* = 4.044 and 8.254), so only the no location priors run is shown. The low Δ*K* at *K* = 2 and large standard deviations around *K* = 2-20 (Wen and Pritchard 2003; Latch et al. 2006) suggest that the optimal *K* is 1. Error bars are standard deviations around each likelihood (Ln P(D); grey squares) based on 10 iterations per *K*.
